# A New Method for Xenogeneic Bone Graft Deproteinization: Comparative Study of Radius Defects in a Rabbit Model

**DOI:** 10.1371/journal.pone.0146005

**Published:** 2015-12-31

**Authors:** Pengfei Lei, Rongxin Sun, Long Wang, Jialin Zhou, Lifei Wan, Tianjian Zhou, Yihe Hu

**Affiliations:** 1 Department of Orthopedics, Xiangya Hospital, Central South University, Changsha, China; 2 Department of Orthopedics, The Sixth Affiliated Hospital of Xinjiang Medical University, Urumqi, China; 3 Department of Orthopedics, Thoracic hospital of Hunan province, Changsha, China; 4 Department of Orthopedics, Ningxiang People’s Hospital, Ningxiang, China; 5 Department of Orthopedics, The First People’s Hospital of Shenzhen, Shenzhen, China; Medical University of South Carolina, UNITED STATES

## Abstract

**Background and Objectives:**

Deproteinization is an indispensable process for the elimination of antigenicity in xenograft bones. However, the hydrogen peroxide (H_2_O_2_) deproteinized xenograft, which is commonly used to repair bone defect, exhibits limited osteoinduction activity. The present study was designed to develop a new method for deproteinization and compare the osteogenic capacities of new pepsin deproteinized xenograft bones with those of conventional H_2_O_2_ deproteinized ones.

**Methods:**

Bones were deproteinized in H_2_O_2_ or pepsin for 8 hours. The morphologies were compared by HE staining. The content of protein and collagen I were measured by the Kjeldahl method and HPLC-MS, respectively. The physical properties were evaluated by SEM and mechanical tests. For *in vivo* study, X-ray, micro-CT and HE staining were employed to monitor the healing processes of radius defects in rabbit models transplanted with different graft materials.

**Results:**

Compared with H_2_O_2_ deproteinized bones, no distinct morphological and physical changes were observed. However, pepsin deproteinized bones showed a lower protein content, and a higher collagen content were preserved. *In vivo* studies showed that pepsin deproteinized bones exhibited better osteogenic performance than H_2_O_2_ deproteinized bones, moreover, the quantity and quality of the newly formed bones were improved as indicated by micro-CT analysis. From the results of histological examination, the newly formed bones in the pepsin group were mature bones.

**Conclusions:**

Pepsin deproteinized xenograft bones show advantages over conventional H_2_O_2_ deproteinized bones with respect to osteogenic capacity; this new method may hold potential clinical value in the development of new biomaterials for bone grafting.

## Introduction

Bone grafting, as one of the preferred therapeutic methods for long bone defects caused by trauma, infection and tumor resection, can be classified into autogenic and xenogenic grafting based on the sources of the implant materials. Although autogenic bone grafting exhibits a distinct therapeutic effect, its application is greatly limited due to the lack of sufficient bone and the secondary injury to donor sites caused by surgery. Therefore, the drawbacks and limitations make it urgent to explore and develop new biomaterials for bone transplantation. Fortunately, bones from other species possess a tissue structure similar to that of human bones. In addition, these bones also exhibit osteoinduction and osteoconduction activities, potentially satisfying the requirements of ideal bone graft substitutes. Xenogenic bone grafting overcomes the obstacles of autogenic grafting, but also faces the potential risks of inducing an immune response and transmitting infectious diseases in patients [1, 2]. Thus, valid strategies to eliminate the antigenicity of xenograft bones are of vital importance in the development of xenogenic bone graft substitutes.

Eliminating the antigens that potentially cause an immune response is a prerequisite for xenogenic bone grafting. MHC-I and MHC-II are the main sources of antigens which induce an immune response in bone grafting, and they are expressed in osteocytes, osteoblasts, osteoclasts and bone marrow cells. While, antigenicity is not detected in trabecular bone and matrix, but it shows different expression levels in osteocytes, osteoblasts, chondrocytes, chondroblasts, bone marrow cells, blood cells and the adipose tissues in bone lacuna. It has been shown that deproteinized bones not only lose their immune reactivity but also retain their osteoinduction and osteoconduction activities [3–5]. The trabecular porous architecture of deproteinized bones acts as a support structure for blood vessels and bone cell expansion which is extremely important for ossification. Methods for bone deproteinization have emerged since Martz *et al*. [6] obtained a deproteinized bovine bone by immersing it in hydrogen peroxide, which has been referred to as Kiel bone. Previous studies showed that the bone reparation materials, such as Bio-oss [4, 7, 8], exhibit a relatively weak clinical effect by a “creeping substitution” process, which limits their application to large bone defects. Liu *et al*. reported an optimized method to improve the osteogenesis ability of processed swine ribs, and a large amount of collagen was preserved [9]. Currently, several chemical and physical methods are utilized to deproteinize the bovine bone scaffolds [3, 10]. Strong oxidant such as H_2_O_2_ or sodium hypochlorite represents the conventional and most popular methods in previous studies [5, 11–15]. However, with the advancement of bone tissue engineering, improved and convenient methods for bovine bone graft deproteinization have been rarely reported.

In this study, we report a new method that utilizes pepsin to deproteinize bovine bones. Comparative studies on the physical, chemical and biological properties of these bones were performed. More importantly, the deproteinized bones showed better therapeutic advantages in the rabbit model of radius defects than bone xenografts treated by conventional method.

## Materials and Methods

### Bovine bone graft preparation

The cancellous bones of the tibias of 2- to 3-year-old Charolais cattle were collected from private slaughterhouses in Zhuzhou (A1-3 small industry promotion district of Tianyuan district, Zhuzhou City, Changsha, China. Geographic coordinates: Lat.27.845°Long.113.091°), by Hengtian Biotechnology Co. Ltd (Zhuzhou, China), then removed soft tissue, bone marrow and cartilage. The bones were cut into cuboid-shaped sticks with dimensions of 0.5 cm × 0.5 cm × 3 cm. The bone sticks were then defatted in a Soxhlet extractor by the petroleum ether extraction method. For hydrogen peroxide deproteinization, the bone sticks were immersed in 30% hydrogen peroxide solution for 8 hours, followed by washing repeatedly with double distilled (ddH_2_O) water. The bones were allowed to dry before use. For the pepsin deproteinization procedure, the bones were digested by 0.3 mg/mL pepsin (Sigma, St. Louis, MO, USA) dissolved in pH 2 phosphate buffer (1.97 g NaH_2_PO4, 1.20 mL H_3_PO4 in 100 mL ddH_2_O) at 25°C for 8 hours. The reactions were terminated by adjusting pH to 9 with NaOH to deactivate the pepsin. After 15 min, the bone sticks were washed repeatedly with ddH_2_O for 30 min and allowed to dry before use.

### Evaluation of collagen I by high-performance liquid chromatography-mass spectrometry (HPLC-MS)

The contents of type I collagen was determined via HPLC-MS by assessing the content of hydroxyproline (Hyp). The chromatographic column selected for analysis was a Luna 5 μ C18 (2) 100A, 150 mm × 2.0 mm, 5 μm column, with nicotinamide (Sigma) as the internal standard, 2% formic acid dissolved in H_2_O as the mobile phase, and acetonitrile as the solid phase (mobile phase: solid phase = 60: 40). The flow rate was 20 μL/min. The MS operating conditions were as follows: ionization mode: ES+, capillary voltage: 3.00 kV, cone voltage: 30.00 V, ion source temperature: 100°C, desolvation gas temperature: 350°C, cone gas flow rate: 50 L/h, desolvation gas flow rate: 400 L/h, ion energy voltage 1: 0.5, ion energy voltage 2: 3.0. The collision pressure was 3.82e-3. The standard curve for hydroxyproline was prepared by the double dilution method with a starting dilution of 497 μg/mL, the following equation was fitted to the prepared standard curve for Hyp: Y = 0.113069 × X ± 0.00491671 (Y: the area ratio of Hyp to nicotinamide in chromatomaps, X: the concentration of Hyp).

### Protein content determination

The protein content of samples in each group was determined by the Kjeldahl method according to the Official Methods and Recommended Practices of AOAC International.

### Determination of pore size and porosity

The pore size was determined by scanning electron microscopy (SEM). Images were captured from multiple sites, and pore diameters were determined by calculating the average values obtained for entire samples of each group. For porosity calculation, the weight of bone sticks before (W1) and after (W2) wax impregnation were compared, the volume after wax impregnation was V1, and the pore volume (V2) was calculated using the equation V2 = (W2-W1)/wax density. Thus, the porosity was obtained by V2/V1.

### Biomechanics evaluation

The biomechanics were tested by an 810 Material Test System (MTS, USA), the samples were selected from each group randomly. The compression load was determined by compression testing (compression speed: 1 mm/min), and the bending load was determined by central-point loading test (span length: 25 mm, bending speed: 1 mm/min).

### *In vitro* cellular affinity assay

The graft materials were cut into small pieces with the dimension of 9 mm × 9 mm × 2 mm and were sterilized. 10000 osteoblasts (MC3T3-E1, provided by the cell bank of Xiangya Hospital of Central South University) were seeded in each graft material, and then placed into a 24-well plate. The graft materials and cells were cultured in a CO_2_ incubator at 37°C for 7 days. The affinity between cells and graft materials were visualized by SEM.

### Alkaline Phsphatase (AKP) assay

The activity of AKP was determinded by commercial kit purchased from Nanjing Jiancheng biotechnology Inc. (Nanjing, China). The absorbance at 520 nm was measured via a spectrophotometer. The AKP activity was normalized by total protein in cell supernatant (determined with BCA protein assay kit, Kangwei, China) and thus expressed as μmol / mg protein. The experiments were repeated for 6 times.

### Calcium deposits

Osteoblast were seeded in each graft material and incubated for 12 and 28 days. The content of calcium deposited was quantified by the Beckman Coulter AU680 auto-analyzer (Beckman Coulter Inc., USA).

### Surgical procedure for radius defect model

Seventy-five New Zealand rabbits from the experimental animal center of the Third Xiangya Hospital of Central South University were anesthetized by administering 3% pentobarbital intravenously. Incisions from the anterolateral of the radius were made, and the muscles and subcutaneous tissues were blunt-dissected to dissociate the radius. 10 mm long bone defects were created surgically, and the periostea in the defect area were completely removed. The rabbits were divided into five groups (*n* = 15 in each group), and then different graft materials were implanted. The defects were not filled with any material in control group, the contralateral radius were dissected and used for autograft group, the medicinal bone matrix (Xiaobo biotechnology, Shanghai, China) for bone matrix group, and the H_2_O_2_ or pepsin deproteinized bovine bones for H_2_O_2_ group and pepsin group, respectively. All surgical procedures were performed under sterile conditions. After implantation, the rabbits were housed separately and treated with 800,000 U/d penicillin intramuscularly for three days. Five rabbits in each group were sacrificed at 4, 8 and 12 weeks after surgery. X-ray (Phillips) and micro-CT (ZKKS-MCT-Sharp III, Zhongkekaisheng, Guangzhou, China) analysis were used to evaluate the differences among groups. The X-ray results were scored by Lane-Sandhu grading [16]. The results of micro-CT were analyzed by ZKKS-MCT-Sharp III 3.0 software. All animal experiments were carried out in accordance with animal welfare guidelines and approved by the Institutional Animal Care and Use Committee (IACUC) of Central South University (approval no. 20130028).

### Histology

After the X-Ray and micro CT analysis, the samples were dissected and immersed in 10% a formaldehyde solution, and then decalcification was carried out by immersing in 1 mol/L HCl for 12 h. The samples were subsequently dehydrated and embedded in paraffin. Hematoxylin-eosin staining was performed and observed under light microscopy.

### Quantitative RT-PCR

Total RNA was isolated from bone tissue using TRIzol reagent (Invitrogen, USA) and was then transcribed to cDNA by a reverse transcription kit from Promega (Madison, WI, USA). The synthesized first cDNA strand were then amplified by a GOTaq SYBR Green master mix (Promega, Madison, WI, USA) in a 7500 Fast Real-Time PCR System (Applied Biosystems, Foster City, CA, USA). The specific PCR primers for IgG: sense 5’- ACGGCGTGGAGGTGCATAATG -3’ and antisense 5’- CGGGAGGCGTGGTCTTGTAGTT -3’. β-actin was used as the internal control.

### Flow cytometry

CD4+/CD8+ T cells were isolated by a CD4+/CD8+ T cell Enrichment Cocktail (Stemcell, Vancouver, BC) according to the the supplier’s instructions. The cells were then stained with FITC conjugated anti-CD4 antibody (Serotec, Indianapolis, IN, USA) and anti-CD8 antibody (GeneTex, GeneTex, Irvine, CA, USA), respectively. The labeled cells were analyzed on a BD FACSVerse™ flow cytometry system (BD Biosciences, Franklin Lakes, NJ, USA).

### Data analysis

Data were expressed as means ± SD. SPSS 15.0 software was used to analyze the data. Differences between two groups were determined by student’s t-test; differences among three or more groups were determined by one-way ANOVA; comparisons between groups were carried out by the LSD t-test. *P*<0.05 was considered to be statistically significant.

## Results

### Morphological comparisons of deproteinized xenograft bones and non-deproteinized xenograft bones

**Fig 1:**
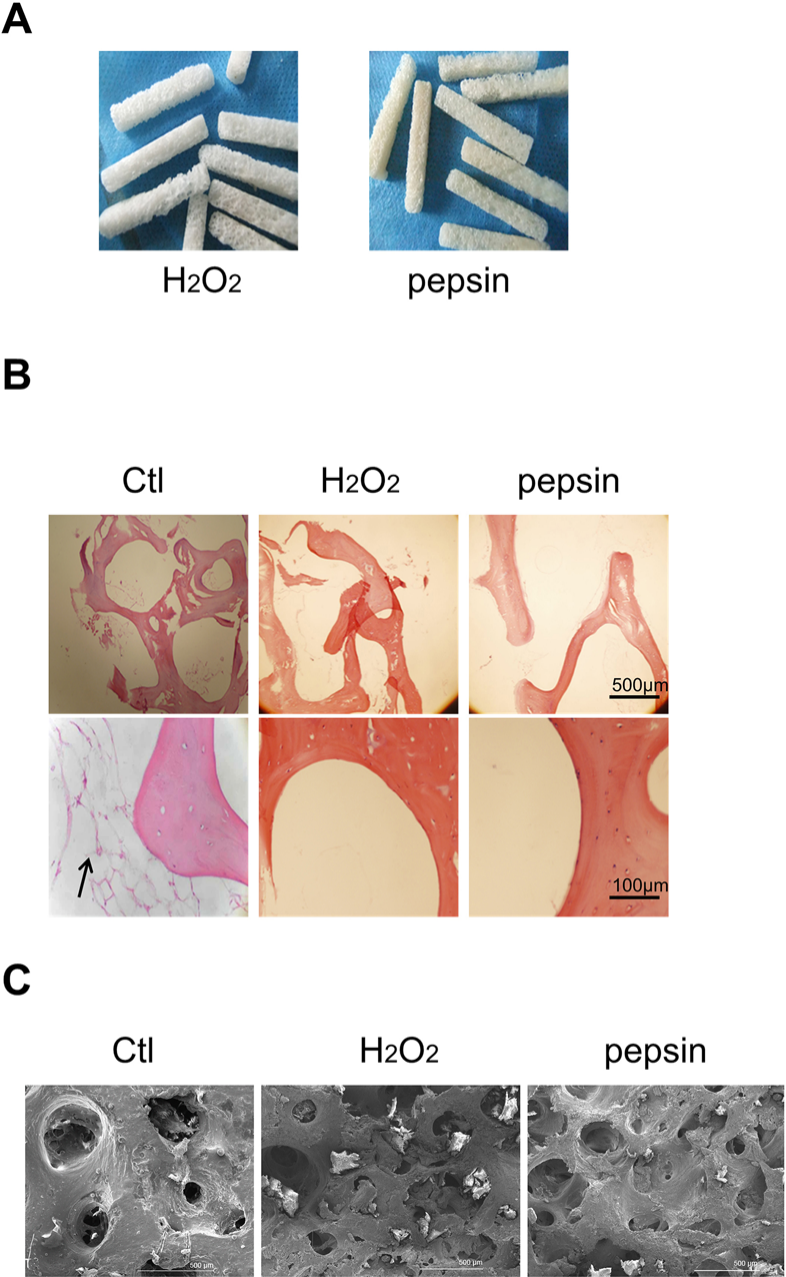
The morphologies of xenograft bones deproteinized by different methods. (A) The macroscopic view of H_2_O_2_ and pepsin deproteinized bovine cancellous bones. (B) The microscopic view (40×: upper panel, 200×: bottom panel) of Ctl, H_2_O_2_ and pepsin deproteinized bovine cancellous bones. The deproteinized bones were decalcified prior to paraffin section and HE staining, the arrow indicates adipose tissue. (C) SEM images of Ctl, H_2_O_2_ and pepsin deproteinized bovine cancellous bones. Ctl: non-deproteinized fresh bones.

The characteristics of the xenograft bones treated by two different deproteinization methods were evaluated. As shown in Fig 1A, there were no distinct macroscopic changes, except for the slight yellow of the pepsin-treated bones. The deproteinized bones possessed an interconnected porous structure, and no soft tissues were attached. Cell debris and adipose tissues were observed in the fresh bone tissues but not in the deproteinized bones (Fig 1B). Moreover, both the H_2_O_2_- and pepsin-treated bovine bones showed a regular collagen structure. Pore size and porosity were analyzd by SEM. From Fig 1C and Table 1, the H_2_O_2_ and pepsin deproteinized bones exhibited a smaller pore size and greater porosity compared with those of the samples in the non-deproteinized control group. Furthermore, the pore size and porosity in pepsin group were smaller than that in H_2_O_2_ group, but the differences were not significant.

**Table 1:** The mean value of pore size and porosity in each groups.

Groups	H_2_O_2_	Pepsin	Ctl (non-deproteinized)
**Pore size (μm)**	512^*^	477^*^	799
**Porosity (%)**	45.02^*^	43.08^*^	35.33

Compared with Ctl,

^*^ *P* < 0.05.

### Biochemical and biomechanical analysis of deproteinized xenograft bones

**Fig 2:**
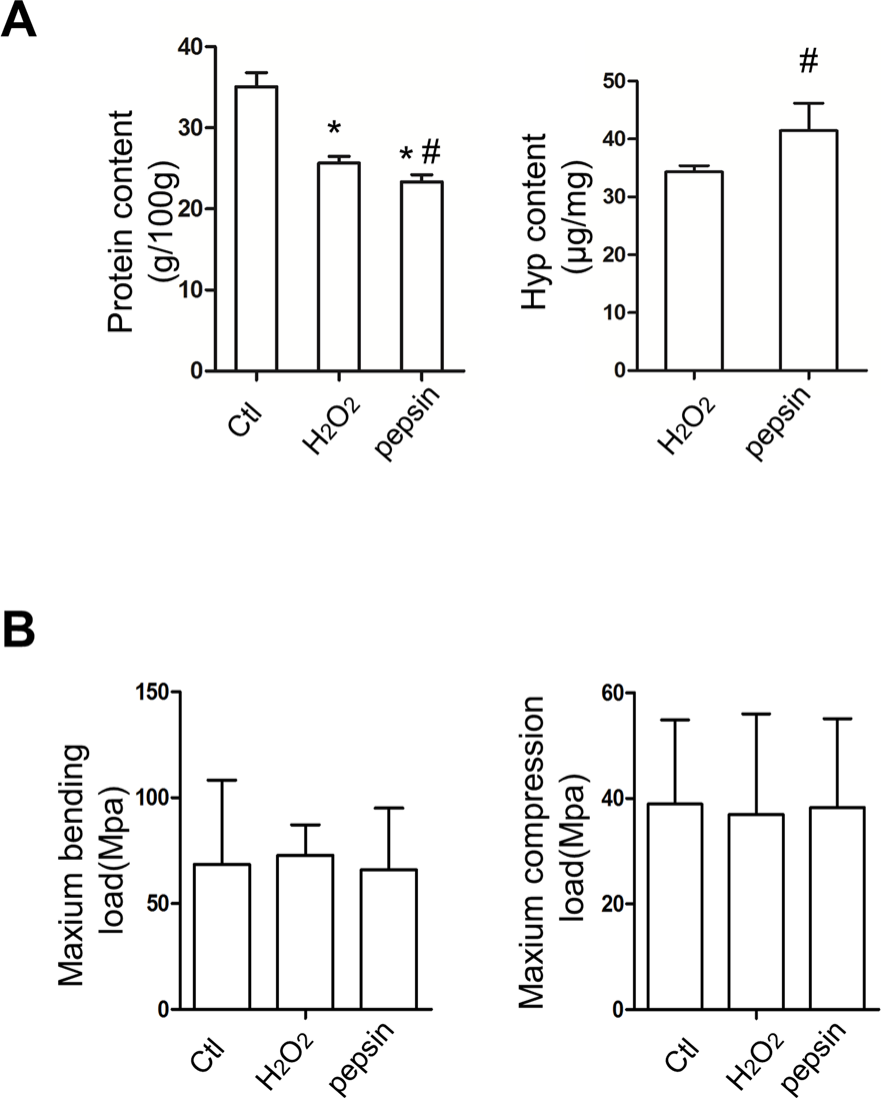
The biochemical and biomechanical analysis of H_2_O_2_ and pepsin deproteinized xenograft bones (*n*≥5) (A) The protein content (g/100g) and hydroxyproline content (μg/mg) of bovine cancellous bones in different groups. Bones were ground and subjected to the Kjeldahl method and HPLC-MS for protein content and hydroxyproline content, respectively. (B) The biomechanical properties of each group. No differences were observed in maximum compression load (Mpa) and bending load (Mpa). Compared with Ctl, ^*^ *P* < 0.05. Compared with H_2_O_2_, ^#^ *P* < 0.05.

Deproteinization is an indispensable process for the bone xenografts. With the Kjeldahl method, the two deproteinization methods resulted in a significant decrease in protein content, and the protein content in pepsin-treated xenograft bones was lower than that in H_2_O_2_-treated bones (Fig 2A). The content of collagen is related to the biomechanical properties. Hydroxyproline is an imino acid typical of collagen. Therefore, the determination on hydroxyproline can reflect the content of collagen. As shown in Fig 2A, the hydroxyproline level was higher in pepsin-treated bones than in H_2_O_2_-treated bones. In biomechanical tests, the compression load and bending load were evaluated (Fig 2B). The results from this study indicated the differences among the three groups were not significant, which suggested the deproteinization procedure did not affect the biomechanical characteristics of xenograft bones.

### *In vitro* cellular affinity assay, AKP activity and calcium deposits

**Fig 3:**
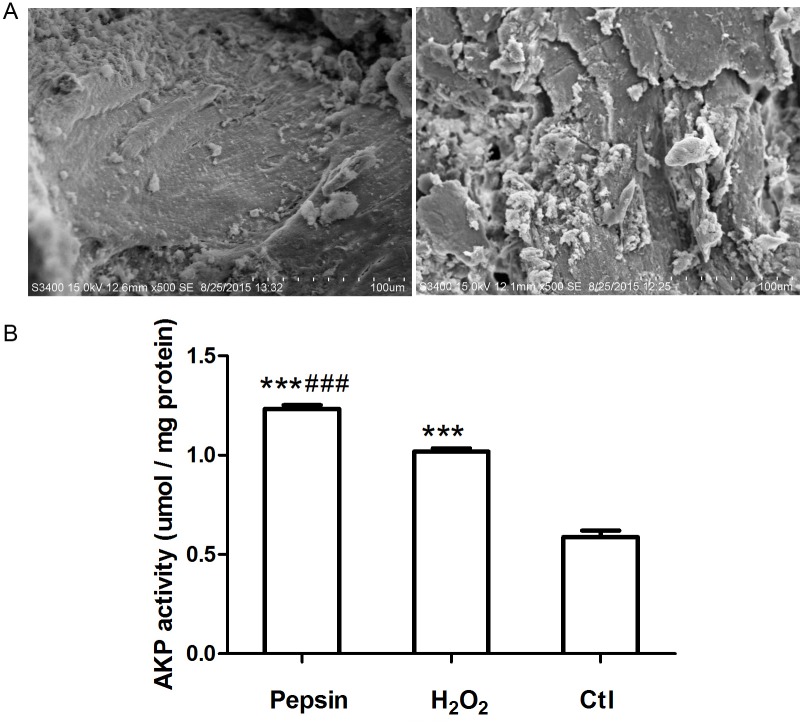
(A) *In vitro* cellular affinity assay showed high cell proliferation of osteoblasts in pepsin treated bones (left) and H_2_O_2_ treated bones (right) (magnification × 550). (B) The AKP activity was significantly increased in osteoblasts and pepsin treated bones co-culture system. Compared with Ctl, ^***^ *P* < 0.001. Compared with H_2_O_2_, ^###^ *P* < 0.001.

**Fig 4:**
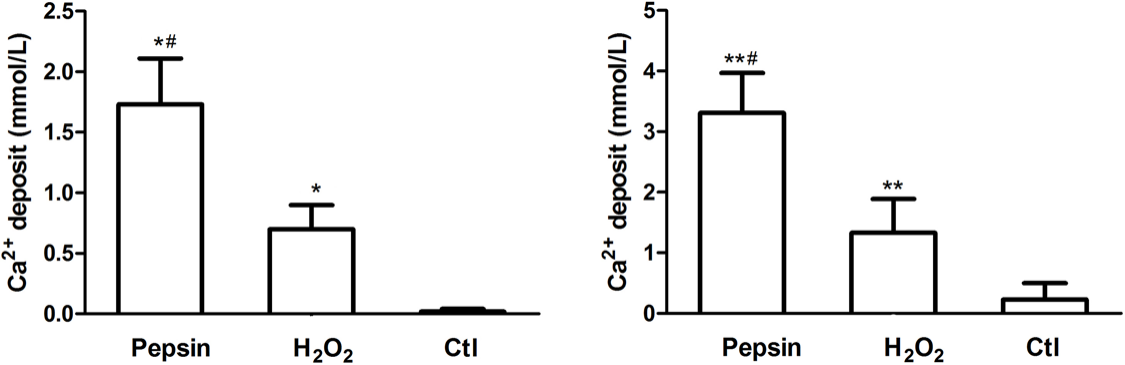
Quantification of calcium deposits of osteoblast cultured on pepsin treated bones, H_2_O_2_ treated bones and non-deproteinized fresh bones on day 14 (left) and day 28 (right). Compared with Ctl, ^*^ *P* < 0.05. Compared with H_2_O_2_, ^#^ *P* < 0.05.

*In vitro* cellular affinity and AKP activity of pepsin and H_2_O_2_ deproteinized bones were further compared. As shown in Fig 3A, there were no distinct differences in cell adhesion between pepsin treated bones and H_2_O_2_ treated bones. While AKP activity was increased in osteoblasts and pepsin treated bones co-culture system, and it was significantly higher than that in H_2_O_2_ treated bones and non-deproteinized fresh bones (Fig 3B). Similarly, calcium deposits (shown in Fig 4) of osteoblasts in pepsin treated bones were significantly higher than that in H_2_O_2_ treated bones and non-deproteinized fresh bones at 14 days. The contrast is even clear at 28 days.

### Pepsin deproteinized xenograft bones show improved osteogenic performance *in vivo*

**Fig 5:**
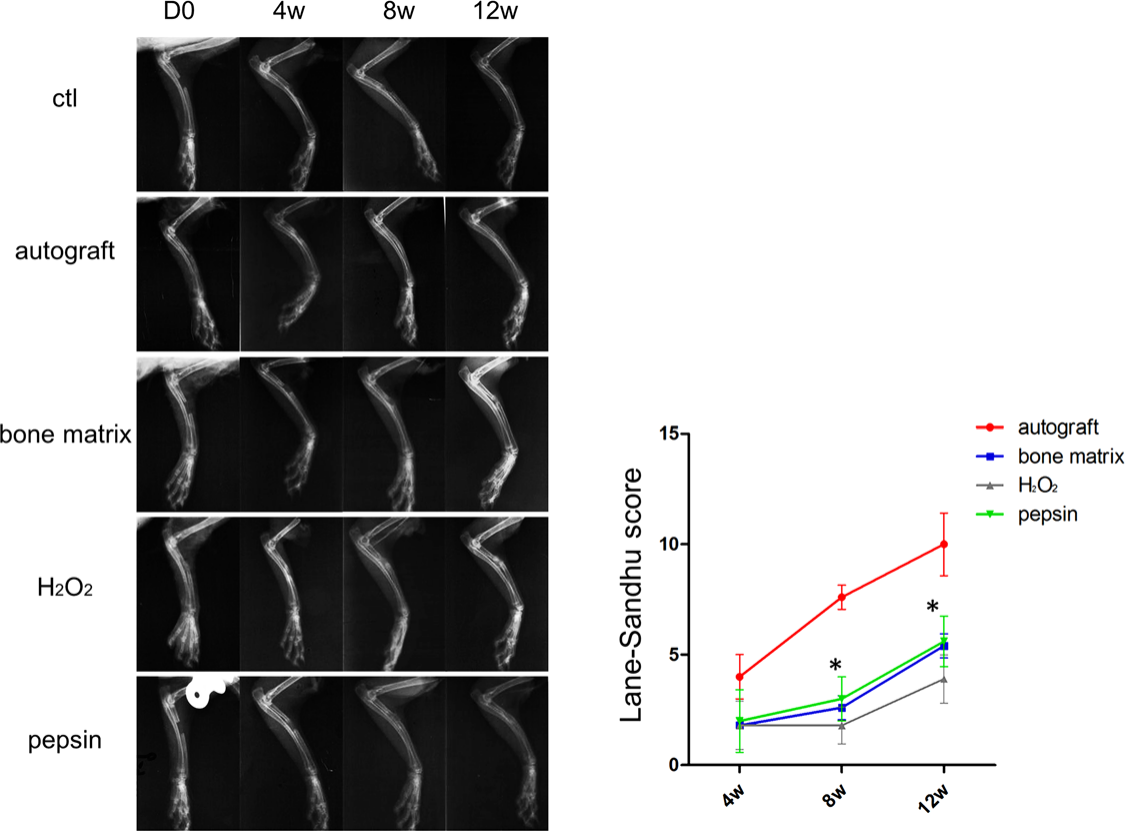
The X-ray analysis showed the osteogensis performance of different graft materials (*n* = 5). Different graft materials were implanted in defect sites, the X-ray images were taken 0 day (D 0), 4 weeks (4 w), 8 weeks (8 w) and 12 weeks (12 w) after bone grafting, respectively. The X-ray results were scored by Lane-Sandhu grading method and the statistical data were shown in the right panel. Compared with H_2_O_2_, ^*^ *P* < 0.05.

To determine whether the two deproteinization methods yielded different performance *in vivo*, the osteogenesis experiment on a rabbit model was performed. The upper region of each rabbits’ radius was surgically removed to establish an animal model of radius defects. Then, autograft, medicinal inductive bone matrix, H_2_O_2_ deproteinized bovine bones and pepsin deproteinized bovine bones were implanted into the defected site. The entire osteogenesis process were traced by X-ray, micro-CT and histological examination. As shown in Fig 5, the results of the X-ray analysis clearly displayed the healing process of each group. In non-transplanted control group, the bone marrow cavities were closed completely, without bony callus. For autograft group, the bone marrow cavities began to become interconnected at 4 weeks post-surgery, the cortical bones were well remodeled. In the bone matrix transplanted group, fracture lines were clearly presented, the high-density bone calluses were connected with the broken end of the fracture bone after 12 weeks, and bone outlines were formed on the outer side of the bone calluses. In the H_2_O_2_ deproteinized group, low-density bone calluses were observed from 4 weeks to 12 weeks post-surgery, the bone outline was scarcely observed, whereas the bone fracture lines were still visible at 12 weeks post-surgery. Surprisingly, the pepsin deproteinized group showed tremendous improvement during the healing process, the density of bone callus was increased significantly from 8 weeks to 12 weeks post-surgery. Furthermore, the bones were well connected, and the bone fracture lines were undetectable. The bone formation scores were then further evaluated according to the Lane-Sandhu score standard. The statistical data showed that the Lane-Sandhu score was similar between the pepsin deproteinized group and the medicinal bone matrix transplanted group, but it was significantly higher than that of the H_2_O_2_ deproteinized group, which revealed that the pepsin deproteinization improved the osteogenesis process.

### Pepsin deproteinized xenograft bones improve bone quantity and quality

**Fig 6:**
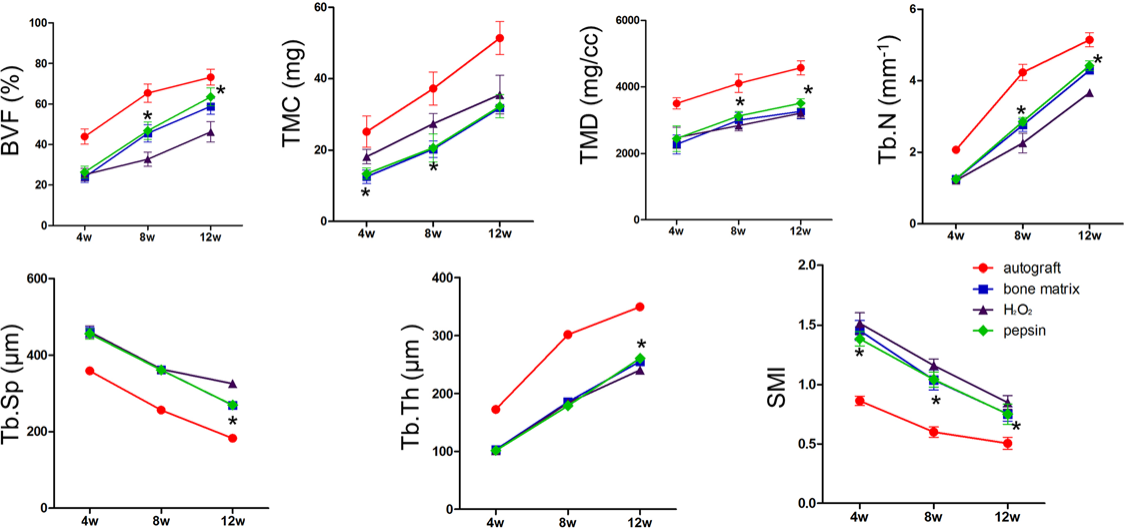
The micro-CT analysis showed the bone quantity and quality in different groups (*n* = 5). The following parameters were measured at the indicated time points: bone volume fraction (BVF), tissue mineral content (TMC), tissue mineral density (TMD), trabecular number (Tb.N), trabecular separation (Tb.sp), trabecular thickness (Tb.th) and structure model index (SMI). Compared with H_2_O_2_, ^*^ *P* < 0.05.

To evaluate the quantity and quality of newly formed bones, micro-CT assessment was conducted (S1 Fig). The bone volume fraction (BVF), tissue mineral content (TMC), tissue mineral density (TMD), trabecular number (Tb.N), trabecular separation (Tb.sp), trabecular thickness (Tb.th) and structure model index (SMI) were measured. These parameters were established to reflect the architecture of bones [17–20]. As shown in Fig 6, the autograft group showed dominant advantages over other groups with respect to BVF, TMC, TMD, Tb.N and Tb.th, whereas the Tb.sp and SMI were the lowest among all the groups. All the parameters were not significant difference between the pepsin group and the bone matrix group. Furthermore, higher levels of BVF, TMD Tb.N and Tb.th were observed in the pepsin group compared with those of the H_2_O_2_ group from 8 to 12 weeks post-surgery. And Tb.sp and SMI levels were lower in the pepsin group than the H_2_O_2_ group at 12 weeks post-surgery, but without significant difference in TMC values between them.

### Pepsin deproteinized xenograft bones improve the maturation of osteocytes

**Fig 7:**
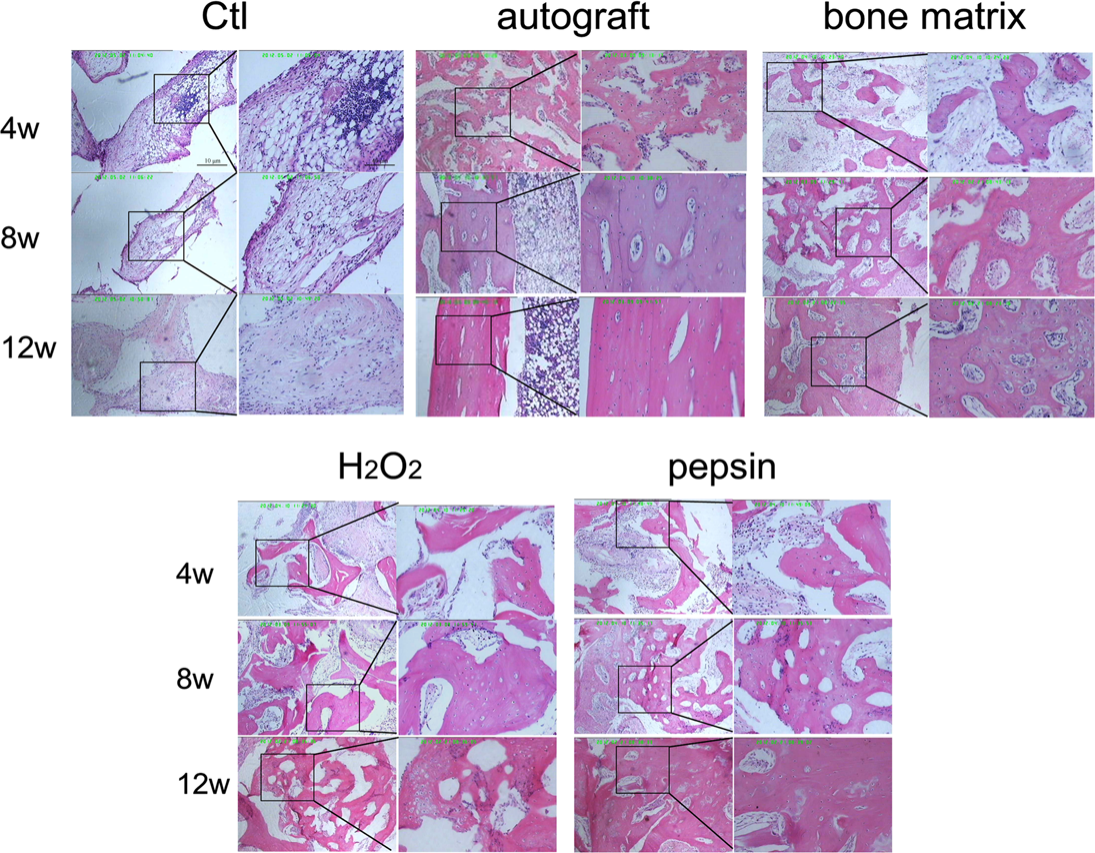
The histological analysis of bones in the defect sites of different groups. The defect sites of radius in each group were dissected and subjected to HE staining. The pictures on the left side of each group represent the low power microscopic views (40 ×); the magnified views of boxed areas (200 ×) were shown on the right side of each group.

Histological analysis was performed to detect the inflammatory cell infiltration and the formation of trabecular as well as connective tissue. As shown in Fig 7, inflammatory cells were observed over the entire period after surgery in the non-transplanted control group. The connective tissues were clearly observed, and no trabecular bone was detected in defect sites. In the autograft group, the inflammatory cells were gradually disappeared from 4 to 12 weeks post-surgery. Moreover, the osteocytes were mature, a large number of trabecular bones were formed 4 weeks post-surgery and lamellar bones were observed from 8 to 12 weeks post-surgery. The microscopy results obtained for the pepsin group were very close to those obtained for the bone matrix group, trabecular bones began to form at 4 weeks after surgery, a large amount of osteocytes were observed from 8 weeks post-surgery, and woven bones gradually transformed into lamellar bones from 8 to 12 weeks after surgery. Overall, the osteocytes became mature, and inflammatory cells were seldom observed. However, in the H_2_O_2_ group, although there were many newly formed trabecular bones, the number of inflammatory cells in defect sites was greater than the pepsin group at 8 weeks post-surgery. In addition, most of the newly formed bones were composed of woven bones, and lamellar bones were not clearly observed at 12 weeks post-surgery. These results suggested that pepsin deproteinized xenograft bones show a better effect on osteocyte maturation than H_2_O_2_ deproteinized xenograft bones.

### Pepsin deproteinized xenograft bones reduces immune-reactivity upon bone grafting

**Fig 8:**
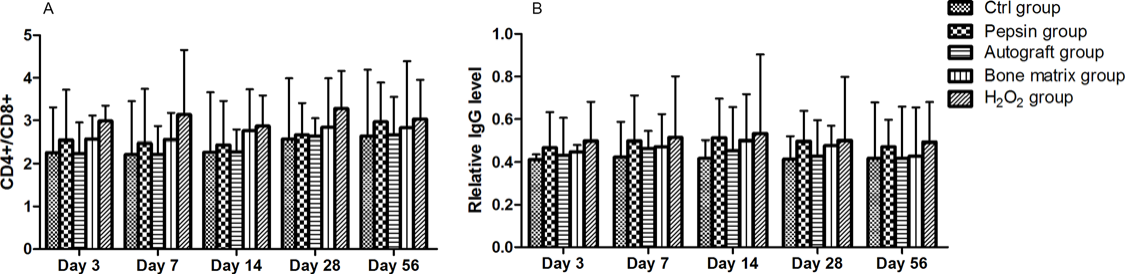
Pepsin deproteinized xenograft bones reduces immune-reactivity upon bone grafting. The activity of cellular immunity (CD4+/CD8+ ratio) was significantly higher in H_2_O_2_ group when compared with that in the pepsin group (A); humoral immunity activity (IgG level) was expressed at a lower level at the donor site in the pepsin group (B).

The blood sample and the tissues at the defect site in each rabbit were collected to compare the immunogenicity of different graft materials. CD4+/CD8+ ratio, which reflects the activity of cellular immunity, was significantly higher in H_2_O_2_ group compared with pepsin group (Fig 8A). In addition, IgG, which reflects humoral immunity activity, was expressed at a lower level at the donor site (Fig 8B). These results strongly suggested that pepsin deproteinized xenograft exhibited less immunogenicity in the radius bone graft model. (All the data in the result section are available in S1 File.)

## Discussion

In this study, pepsin was used in the deproteinization for xenografting bones. Compared with the conventional H_2_O_2_ deproteinization method, the new method did not result in any detectable biomechanical defects in bovine bones after deproteinization, at the same time, the new pepsin deproteinization method showed a significant improvement in protein removal, and more collagen I was preserved. Furthermore, *in vivo* studies demonstrated that the pepsin deproteinized xenograft bones performed better than those deproteinized by conventional H_2_O_2_ method.

Bones are formed by the regular deposition of inorganic matter within collagen fibers. Collagen I is the main source of organic matter in bone and provides basic tenacity and flexibility, which are essential to the biomechanical properties of bone [21]. Collagen I not only offers scaffolds for bone mineralization, but also plays crucial roles in the proliferation and adhesion of cells. It has been reported that collagen-modified biomaterials display enhanced bioactivity [22–25], which is in favour of osteocyte proliferation, adhesion and bone tissue regeneration. Moreover, in bone grafting, collagen exerts beneficial effects on osteogenesis coupled with the reduction of immunologic rejection [26–29]. As the component of a good xenogenic material was very similar to the normal human bones, thus the preserve of collagen I in xenograft bones is very important. Cancellous bones contain a large amount of bone marrow cells and abundant protein, the method to eliminate antigenicity is always the key topic in the study of xenograft bone materials. Extensive studies have been conducted to develop methods for bone deproteinization. Strong oxidants such as H_2_O_2_ have been most frequently utilized. H_2_O_2_ could remove antigens efficiently from xenogenic bones, but it also made a destructive effect on inorganic matter and natural bone structures simultaneously, such as the damages to the proper arrangement of Collagen I and promotes collagen fibers decomposition [30]. Besides, increased immunogenicity of collagen after hydroxyl radical modification was also observed [31]. Because of the significant role of collagen I in the osteoinductive function, a more gentle and specific deproteinization method which is able to remove most of proteins and with minimal damage on collagen fiber is in urgent needs. In this study, the collagen content in pepsin treated bones was higher than that in H_2_O_2_ deproteinized bones, which suggested pepsin deproteinization showed less damage on the rigid structure of collagen I. The results preliminarily confirmed that pepsin deproteinized bones were essentially satisfied with the requirements for transplantation.

Osteocytes in defect sites were regenerated from the mesenchymal stem cells (MSCs) that surround muscles and blood vessels [32, 33]. Furthermore, bone morphogenetic proteins (BMPs) secreted by other cells may have the potential to stimulate the differentiation of MSCs to osteocytes. The osteoinduction and osteoconduction activities of xenograft bones were depended on their porous structures [5]. The porous structure not only provided a backbone for ossification but also acted as a shelter for MSC differentiation. *In vitro* experiments confirmed that the pepsin treated bones showed higher affinity with osteoblasts, which indicated that the microstructure of the pepsin treated bones were favorable for cell migration. The increased AKP activity and calcium deposits in pepsin group both suggested that the osteoconductivity and osteoinductivity were well improved by pepsin.

To differentiate it from the repair of normal bone fracture, the healing process of bone defects is called “gap healing”. During the gap healing process, the migration of osteoblasts is much slower than that of connective tissues. So, the defect sites are more likely to be filled with connective tissues, resulting in bone nonunion. In this study, the osteoinduction ability of pepsin deproteinized bones were evaluated in a rabbit radius defect model. X-ray results showed that pepsin treated bones exhibited stronger osteogenic activity. Advanced micro-CT analysis was introduced to examine the bone micro-architecture [20], and the changes of parameters related to bone trabecular indicated that the amount of newly formed bone in rabbits transplanted with pepsin deproteinized bones was greater than that with H_2_O_2_ deproteinized bone. It should be noted that the TMC in H_2_O_2_ group was significantly higher than that in pepsin group at 4 and 8 weeks post-surgery, this is probably due to the demineralization effect of the low pH reaction buffer of pepsin. Surprisingly, no significant difference was observed at the 12 week, which indicated that the calcification of neo-bone was increased with time. More importantly, lower SMI values in the pepsin group suggested that the trabecular bones adopted a more plate-like shape rather than a rod shape, a structure that resembles that observed in osteoporosis [34, 35]. Thus, it can be concluded that the quantity and quality of regenerated bones in pepsin group are superior than other groups. Meanwhile, the study also confirmed the better and long-termed effect in bone remodeling model.

The local microenvironment is crucial for bone regeneration. Appropriate local immune cells could stimulate the differentiation of bone cells and lead to bone maturation, while excessive inflammation prolonged the healing process [36, 37]. In this study, fewer inflammatory cell infiltrations and less immunologic reaction were observed in pepsin group than that in H_2_O_2_ group. Moreover, unlike the newly formed bones in H_2_O_2_ group, those in pepsin group were mature bones. These results were consistent with the biochemical analysis, pepsin treated bones preserved more collagen and less protein than H_2_O_2_, which induced a lower immune response and higher osteoinduction activity.

In conclusion, the new pepsin method for the deproteinization of xenogenic bones presented a better therapeutic effect in the rabbit model of radius defects. The findings provided a new method for the development of new economical xenograft materials with maximum biocompatibilities and bioactivities. The clinical validity will be investigated further in future studies.

## Supporting Information

S1 Fig The quantity and quality of newly formed bones in different groups were assessed by Micro-CT. (TIF)

S1 File The raw data in the result section. (DOCX)
