# Supplementary figures and images for: A New Method for Xenogeneic Bone Graft Deproteinization: Comparative Study of Radius Defects in a Rabbit Model
Source: PLoS One. 2015 Dec 31;10(12):e0146005. doi: 10.1371/journal.pone.0146005 (PMC4699924; doi:10.1371/journal.pone.0146005)

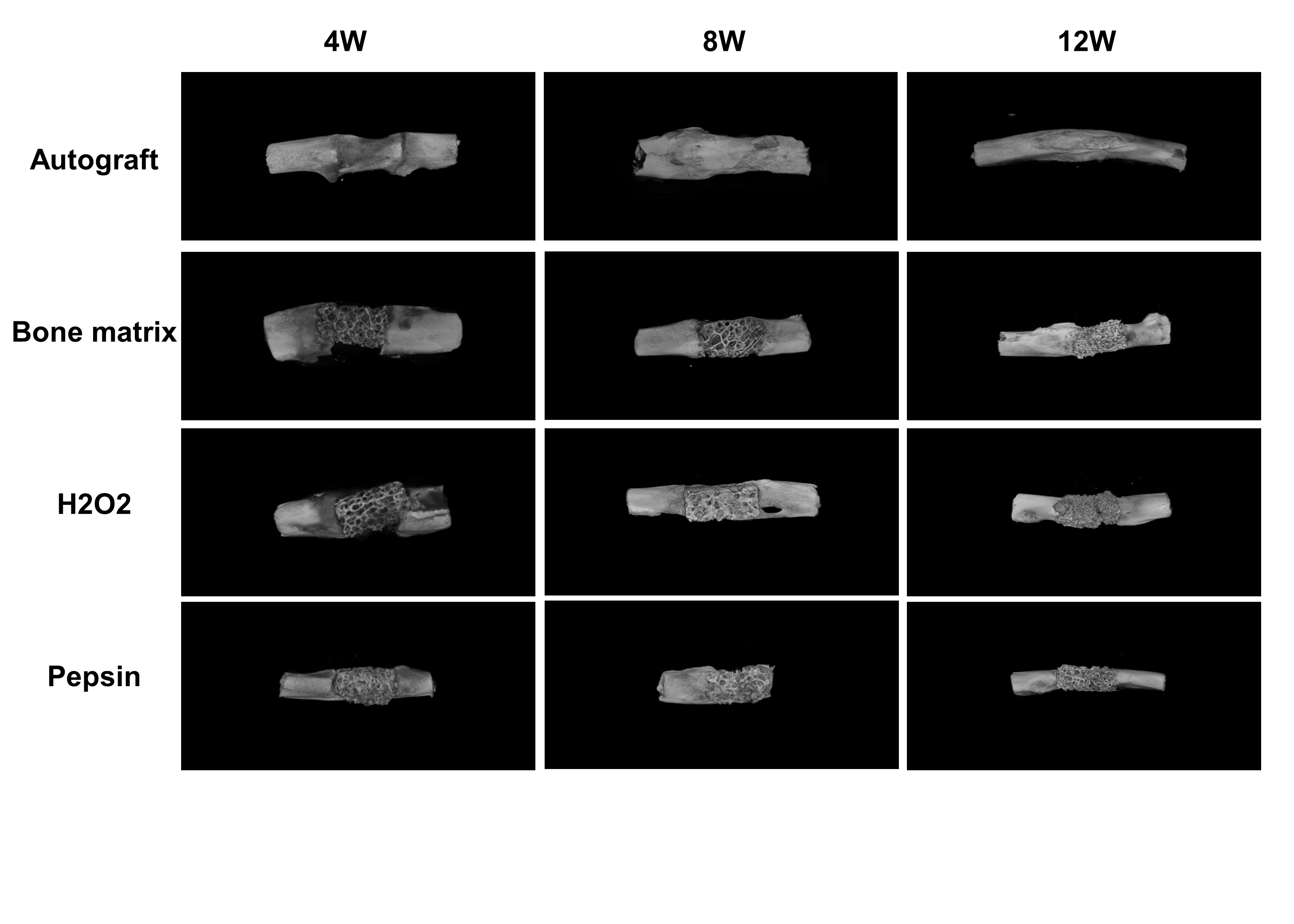

Supplement: S1 Fig — (TIF) [file pone.0146005.s001.tif]
