# Supplementary material for: A New Method for Xenogeneic Bone Graft Deproteinization: Comparative Study of Radius Defects in a Rabbit Model
Source: PLoS One. 2015 Dec 31;10(12):e0146005. doi: 10.1371/journal.pone.0146005 (PMC4699924; doi:10.1371/journal.pone.0146005)
Supplement: S1 File — (DOCX) [file pone.0146005.s002.docx]

**All the data in the result section**

**The data used in Fig 2**

Table 1 The protein content (g/100g) and hydroxyproline content (μg/mg) of bovine cancellous bones in different groups.

| Groups | Protein content (g/100g) | Hydroxyproline content (μg/mg) |
| --- | --- | --- |
| H_2_O_2_ | 25.65±0.82 | 34.3143±1.0789 |
| Pepsin | 23.34±0.86 | 41.4514±2.3416 |
| Ctl | 35.08±1.72 |  |

Table 2 The maximum compression load (Mpa) and bending load (Mpa) in different groups.

| Groups | The maximum bending load (Mpa) | The maximum compression load (Mpa) |
| --- | --- | --- |
| Ctl | 68.44±39.91 | 38.92±15.97 |
| H_2_O_2_ | 72.82±14.38 | 36.95±19.06 |
| Pepsin | 65.93±29.18 | 38.29±16.85 |

**The data used in Fig 3**

Table 3 The AKP activity in osteoblasts.

|  | 1 | 2 | 3 | | 4 | | 5 | 6 | |
| --- | --- | --- | --- | --- | --- | --- | --- | --- | --- |
| Pepsin | 1.2085 | 1.2335 | 1.2332 | | 1.2182 | 1.3179 | | | 1.1900 |
| H_2_O_2_ | 0.6105 | 0.7343 | 0.5146 | 0.5506 | | | 0.5352 | | 0.5822 |
| Ctl | 0.9686 | 1.0772 | 1.0046 | | 1.0053 | 1.0459 | | | 1.0097 |

**The data used in Fig 4**

Table 4 Quantification of calcium deposits of osteoblast cultured on pepsin treated bones, H_2_O_2_ treated bones and non-deproteinized fresh bones on day 14 and day 28.

|  |  | 1 | 2 | 3 | 4 | 5 | 6 |
| --- | --- | --- | --- | --- | --- | --- | --- |
| Day 14 | Pepsin | 2.16 | 1.56 | 1.47 |  |  |  |
|  | H_2_O_2_ | 0.48 | 0.87 | 0.76 |  |  |  |
|  | Ctl | 0.01 | 0.04 | 0 | 0 | 0.04 | 0 |
| Day 28 | Pepsin | 4.06 | 2.87 | 2.99 |  |  |  |
|  | H_2_O_2_ | 0.74 | 1.85 | 1.41 |  |  |  |
|  | Ctl | 0.16 | 0.18 | 0.11 | 0.03 | 0.12 | 0.76 |

**The data used in Fig 5**

Table 5 The X-ray results were scored by Lane-Sandhu grading method.

|  | Autograft | Bone matrix | H_2_O_2_ | Pepsin |
| --- | --- | --- | --- | --- |
| 4 weeks | 4.0000±1.0000 | 1.8000±1.0954 | 1.8000±1.0954 | 2.0000±1.4142 |
| 8 weeks | 7.6000±0.5477 | 2.6000±0.5477 | 1.8000±0.8367 | 3.0000±1.0000 |
| 12 weeks | 10.0000±1.4142 | 5.4000±0.5477 | 3.8000±1.0954 | 5.6000±1.1402 |

**The data used in Fig 6**

Table 6 The parameters in bone quantity and quality in different groups.

4 weeks after bone grafting

|  | Autograft | Bone matrix | H_2_O_2_ | Pepsin | |
| --- | --- | --- | --- | --- | --- |
| BVF | 43.9310±3.7180 | 23.8398±2.5794 | 25.1637±3.1062 | | 26.3421±2.9274 |
| TMC | 25.1558±4.3631 | 12.5335±1.8718 | 18.1762±2.0542 | | 13.3921±1.6263 |
| TMD | 3506.353±166.573 | 2269.547±282.779 | 2471.542±315.163 | | 2444.351±387.553 |
| Tb.N | 2.0685±0.0443 | 1.2510±0.0842 | 1.2163±0.0447 | | 1.2525±0.0303 |
| Tb.sp | 358.7335±10.7479 | 460.5125±13.4625 | 460.4982±15.8905 | | 455.5409±13.5110 |
| Tb.th | 172.3352±2.7534 | 102.7268±2.7512 | 101.4764±4.1640 | | 101.5875±3.1170 |
| SMI | 0.8629±0.0395 | 1.4545±0.0841 | 1.5149±0.0884 | | 1.3848±0.0602 |

8 weeks after bone grafting

|  | Autograft | Bone matrix | H_2_O_2_ | Pepsin | |
| --- | --- | --- | --- | --- | --- |
| BVF | 65.4195±4.4582 | 45.5079±4.1446 | 32.7229±3.5243 | | 46.8408±4.2744 |
| TMC | 37.2519±4.6108 | 20.2853±2.2879 | 27.4032±2.8420 | | 20.6078±3.9347 |
| TMD | 4109.706±271.016 | 3000.780±231.106 | 2836.640±155.068 | | 3124.495±140.924 |
| Tb.N | 4.2329±0.2273 | 2.7605±0.1724 | 2.2587±0.2732 | | 2.8597±0.1149 |
| Tb.sp | 256.2660±5.6385 | 361.3480±4.6125 | 363.3526±2.5885 | | 361.2012±2.3597 |
| Tb.th | 301.7434±4.6508 | 185.5614±5.0698 | 183.7920±4.1825 | | 178.6813±3.0391 |
| SMI | 0.6000±0.0436 | 1.0385±0.0838 | 1.1608±0.0546 | | 1.0435±0.0633 |

12 weeks after bone grafting

|  | Autograft | Bone matrix | H_2_O_2_ | Pepsin | |
| --- | --- | --- | --- | --- | --- |
| BVF | 73.2116±3.9233 | 58.8338±3.9478 | 46.2864±5.0549 | | 63.5391±4.4408 |
| TMC | 51.4193±4.5743 | 31.8271±2.7462 | 35.5259±5.4042 | | 32.2598±3.2643 |
| TMD | 4575.717±209.721 | 3264.103±222.152 | 3211.990±155.508 | | 3507.892±140.376 |
| Tb.N | 5.1436±0.1933 | 4.2941±0.1007 | 3.6623±0.0789 | | 4.4216±0.1370 |
| Tb.sp | 182.6838±1.8411 | 268.8554±3.0251 | 325.3968±3.3277 | | 269.8022±1.8991 |
| Tb.th | 349.4844±3.9375 | 255.2936±1.8644 | 240.5382±2.3576 | | 260.6295±2.4462 |
| SMI | 0.5058±0.0508 | 0.7519±0.0610 | 0.8485±0.0589 | | 0.7502±0.0849 |

**The data used in Fig 8**

Table 7 Percentage of CD4+, CD8+ and the ratio CD4+/CD8+ in peripheral blood.

| Group | Time after Surgery(days) | CD4+ | CD8+ | CD4+/CD8+ |
| --- | --- | --- | --- | --- |
| Blank group | 0 | 12.55±1.25 | 7.41±0.75 | 1.69±0.72 |
|  | 3 | 19.36±4.17 | 8.55±0.55 | 2.26±1.05 |
|  | 7 | 24.17±1.47 | 11.18±0.51 | 2.21±1.24 |
|  | 14 | 24.25±2.25 | 14.96±0.64 | 2.27±1.39 |
|  | 28 | 24.11±1.44 | 13.37±1.23 | 2.57±1.42 |
|  | 56 | 22.01±2.37 | 13.01±0.88 | 2.64±1.55 |
| Pepsin group | 3 | 22.26±1.64 | 9.53±0.54 | 2.55±1.17 |
|  | 7 | 24.87±1.47 | 12.81±0.60 | 2.48±1.26 |
|  | 14 | 24.62±0.91 | 15.34±0.68 | 2.44±1.02 |
|  | 28 | 29.72±1.10 | 15.54±1.37 | 2.67±0.74 |
|  | 56 | 24.40±0.81 | 13.37±0.64 | 2.97±0.92 |
| Autologous bone group | 3 | 20.03±2.57 | 8.97±0.66 | 2.23±0.72 |
|  | 7 | 23.38±0.91 | 11.43±0.11 | 2.22±0.65 |
|  | 14 | 24.92±1.04 | 14.99±0.57 | 2.28±0.51 |
|  | 28 | 25.33±0.10 | 13.43±0.14 | 2.63±0.42 |
|  | 56 | 23.82±1.68 | 13.27±1.09 | 2.66±0.89 |
| Bone matrix group | 3 | 23.62±0.86 | 9.85±0.23 | 2.57±0.55 |
|  | 7 | 25.73±1.60 | 12.24±0.69 | 2.56±0.62 |
|  | 14 | 25.28±3.19 | 17.20±1.03 | 2.76±0.97 |
|  | 28 | 30.43±2.20 | 14.24±0.18 | 2.84±1.15 |
|  | 56 | 25.84±1.30 | 13.90±0.49 | 2.83±1.56 |
| Hydrogen peroxide group | 3 | 24.82±3.25 | 10.28±0.74 | 2.99±0.36 |
|  | 7 | 26.67±0.94 | 13.07±0.49 | 3.14±1.51 |
|  | 14 | 33.87±0.58 | 18.07±0.21 | 2.87±0.72 |
|  | 28 | 46.79±2.02 | 17.05±0.58 | 3.28±0.88 |
|  | 56 | 41.37±0.42 | 14.98±0.94 | 3.03±0.92 |

Table 8 The humoral immunity activity (IgG level) at the donor site in the pepsin group.

|  | 3 d | 7 d | 14 d | 28 d | 56 d |
| --- | --- | --- | --- | --- | --- |
| Blank group | 0.412±0.024 | 0.423±0.164 | 0.418±0.084 | 0.415±0.105 | 0.419±0.259 |
| Pepsin group | 0.468±0.165 | 0.498±0.213 | 0.513±0.183 | 0.497±0.142 | 0.472±0.125 |
| Autologous bone group | 0.432±0.174 | 0.464±0.082 | 0.454±0.202 | 0.429±0.166 | 0.418±0.241 |
| Bone matrix group | 0.449±0.032 | 0.473±0.151 | 0.501±0.216 | 0.478±0.092 | 0.429±0.226 |
| Hydrogen peroxide group | 0.498±0.183 | 0.514±0.287 | 0.532±0.372 | 0.501±0.298 | 0.493±0.187 |
